# Detection of cervical lymph node metastasis in head and neck cancer patients with clinically N0 neck—a meta-analysis comparing different imaging modalities

**DOI:** 10.1186/1471-2407-12-236

**Published:** 2012-06-12

**Authors:** Li-Jen Liao, Wu-Chia Lo, Wan-Lun Hsu, Chi-Te Wang, Mei-Shu Lai

**Affiliations:** 1 Graduate Institute of Epidemiology and Preventive Medicine, College of Public Health, National Taiwan University, Taipei, Taiwan; 2 Department of Otolaryngology, Far Eastern Memorial Hospital, Taipei, Taiwan; 3 Genomics Research Center, Academia Sinica, Taipei, Taiwan; 4 Center of Comparative Effectiveness Research, National Center of Excellence for Clinical Trial and Research, National Taiwan University Hospital, Taipei, Taiwan

## Abstract

**Background:**

How to properly manage clinically negative neck of head and neck cancer patients is a controversial topic. Research is now directed toward finding a method sensitive enough to bring the risk of occult metastases below 20%. The aim of this review was to compare the diagnostic accuracy of different imaging modalities, including CT, MRI, PET and US, in clinically N0 head and neck cancer patients.

**Methods:**

For this systematic review and meta-analysis, PubMed and the Cochrane Database were searched for relevant original articles published up to May 2011. Inclusion criteria were as follows: articles were reported in English; CT, MRI, PET or US were performed to identify cervical metastases in clinically N0 head and neck squamous cell carcinoma; and data were sufficient for the calculation of true-positive or false-negative values. A bivariate random effect model was used to obtain pooled sensitivity and specificity. The positive and negative test probability of neck metastasis was generated based on Bayesian theory and collected data for different pre-test possibilities.

**Results:**

Of the 168 identified relevant articles, 7 studies fulfilled all inclusion criteria for CT, 6 studies for MRI, 11 studies for PET and 8 studies for US. There was no difference in sensitivity and specificity among these imaging modalities, except CT was superior to US in specificity. The pooled estimates for sensitivity were 52% (95% confidence interval [CI], 39% ~ 65%), 65% (34 ~ 87%) 66% (47 ~ 80%), and 66% (45 ~ 77%), on a per-neck basis for CT, MRI, PET and US, respectively. The pooled estimates for specificity were 93% (87% ~ 97%), 81% (64 ~ 91%), 87% (77 ~ 93%), and 78% (71 ~ 83%) for CT, MRI, PET and US, respectively. With pre-examination nodal metastasis probabilities set at 10%, 20% and 30%, the post-exam probabilities of positive nodal metastasis rates were 47%, 66% and 77% for CT; 27%, 46% and 59% for MRI; 36%, 56% and 69% for PET; and 25%, 42% and 56% for US, respectively. Negative nodal metastasis probabilities were 95%, 89% and 82% for CT; 95%, 90% and 84% for MRI; 96%, 91% and 86% for PET; and 95%, 90% and 84% for US, respectively.

**Conclusions:**

Modern imaging modalities offer similar diagnostic accuracy to define and diagnose clinically N0 neck. Minimizing morbidity and avoiding elective neck dissection is acceptable in some select cases.

## Background

Lymph node status is one of the most important predictors of poor prognosis in head and neck cancers. For patients with clinically positive (cN+) neck lymph node metastasis, modified radical neck dissection is a standard procedure. For patients with clinically negative (cN0) neck, there are two major management strategies, which include elective neck dissection or a “watchful waiting policy”. Cervical lymph node metastasis staged by palpation has been demonstrated to be inaccurate; the rate of occult cervical nodal metastases is at least 30% by simple palpation [1]. To avoid the unnecessary treatment of histologically negative necks, a staging technique must be sensitive enough to reduce the risk of occult metastases to less than 20%, which means a negative predictive value (NPV) of more than 80% [2,3]. Bayesian logic states that the probability of a disease being present given that a test is negative depends on the pre-test probability or the prevalence of the disease, and the sensitivity and specificity of the test can be described by the following formula [4]:

(1) $$Negative\;predictive\;value=specificity*\left(1-prevalence\right)/[specificity*\left(1-prevalence+\left(1-sensitivity\right)*prevalence\right]$$

With the development of modern imaging modalities, the American Joint Committee on Cancer has stated that clinical staging should include physical examination as well as the results of other imaging modalities. Research is now directed toward finding a staging method sensitive enough to bring the risk of occult metastases below 20%. Previous meta-analyses [2,5] compared the diagnostic accuracy of different imaging modalities in neck node evaluation. However, these studies looked at a mixture of cN + and cN0 patients, and no study has focused only on patients with cN0 necks. The aim of this study was to complete a systematic review for the performance of different imaging modalities, i.e., computed tomography (CT), magnetic resonance imaging (MRI), positron emission tomography (PET) and ultrasound (US), in the evaluation of neck lymph node metastasis in clinically N0 head and neck cancer patients.

## Methods

### Literature search

A computerized systematic literature search was performed by one reviewer (Liao LJ). Abstracts were selected for English articles reporting on diagnostic performance in the detection of lymph node metastasis in head and neck cancers. The search strategy used text and relevant indexing to capture the concept of ultrasonography in head and neck cancer patients with N0 neck evaluations.

PubMed (up to May 2011) and CENTRAL (via the Cochrane database up to October 2010) were searched using the following keywords: (a) (Head and Neck Neoplasms[MH]) NOT (Thyroid Neoplasms[MH] OR Esophageal Neoplasms[MH] OR Nasopharyngeal Neoplasms[MH] OR Salivary Gland Neoplasms[MH] OR Melanoma[MH] OR Parathyroid Neoplasms[MH]); (b) Diagnostic imaging[MH]; (c) lymph node[TW] OR neck node[TW]; and (d) sensitivity[TW] OR accuracy[TW]. Human studies with abstracts in English were included. Reviews, letters to the author, comments and case reports were excluded.

From the selected abstracts, two reviewers (Lo WC and Wang CT) separately screened the full text of these potentially eligible articles in which computed tomography (CT), magnetic resonance imaging (MRI), positron emission tomography (PET) and ultrasound (US) were utilized. Reference lists were manually screened for additional relevant articles.

### Inclusion criteria

There were two major study groups according to the clinical node staging of the patient population in each study. The first group included studies of patients who had pathologically positive head and neck cancer and clinically negative cervical lymph nodes (cN0) before the imaging examination. The second group included studies with mixed patient populations (head and neck malignancy with both cN0 and cN+). We included studies with individual patient data available for cN0.

Based on the full text reports, studies were selected if they fulfilled all of the following inclusion criteria: (a) histopathology findings for neck dissection (specimens obtained at surgery) or sufficient follow-up time were used as the reference standard; (b) the primary tumor and lymph node metastases were squamous cell carcinomas (SCCs); and (c) sufficient data were presented to construct a 2 × 2 contingency table (sensitivity and/or specificity with absolute numbers of false positive (FP), false negative (FN), true positive (TP) and true negative (TN) findings) for the imaging modalities compared using the reference standard.

### Exclusion criteria

Studies were excluded if raw data were not presented. We also excluded studies with both cN0 and cN positive patients, where individual data for cN0 patients were not available.

### Quality assessment of primary studies

The Quality Assessment of Diagnostic Accuracy Studies (QUADAS)[6] quality assessment tool was used to evaluate the relevant study design characteristics of each study. This tool and the definitions of the characteristics have previously been fully described [6]. We assigned a design characteristic with a score of 1 if the evaluation criteria were met or 0 if the design characteristic was not preset or was unclear. Each study that met the inclusion criteria was analyzed by two independent reviewers (Wang CT and Lo WC). When there was a discrepancy between the reviewers, a consensus reviewer (Liao LJ) resolved these differences. Difference in the QUADAS score among different modalities was tested using the Kruskal-Wallis test.

### Data analysis/synthesis

The primary outcome for analysis was the diagnostic performance of imaging examinations that detected the neck lymph node metastasis compared with the reference standard of neck dissection specimens. Sensitivities and specificities with a 95% confidence interval (CI) values were reported for individual studies. Pooled sensitivities and specificities of CT, MRI, PET and US in neck lymph node metastasis of cN0 neck from individual studies were calculated using a bivariate random effect model [7]. The random effect model incorporated the heterogeneity of the studies into the analysis of the overall efficacy. The differences in sensitivities and specificities were tested using a bivariate random effect model. Likelihood ratios are metrics that are calculated using a combination of sensitivity and specificity values. The positive likelihood ratio (LR+) is defined as the ratio of sensitivity (1-specificity), whereas the negative likelihood ratio (LR-) is defined as the ratio of specificity (1-sensitivity). When a diagnostic test has absolutely no discriminating ability, both likelihood ratios equal 1. The performance of each diagnostic modality was shown using summary receiver operating characteristic (ROC) curves. The positive and negative post-test probability was generated based on Bayesian theory, and collected data were tabulated for 10%, 20% and 30% pre-test probabilities of cN0 neck. Statistical analyses were completed using STATA version 10.0 (Stata Corp.).

## Results

**Figure 1:**
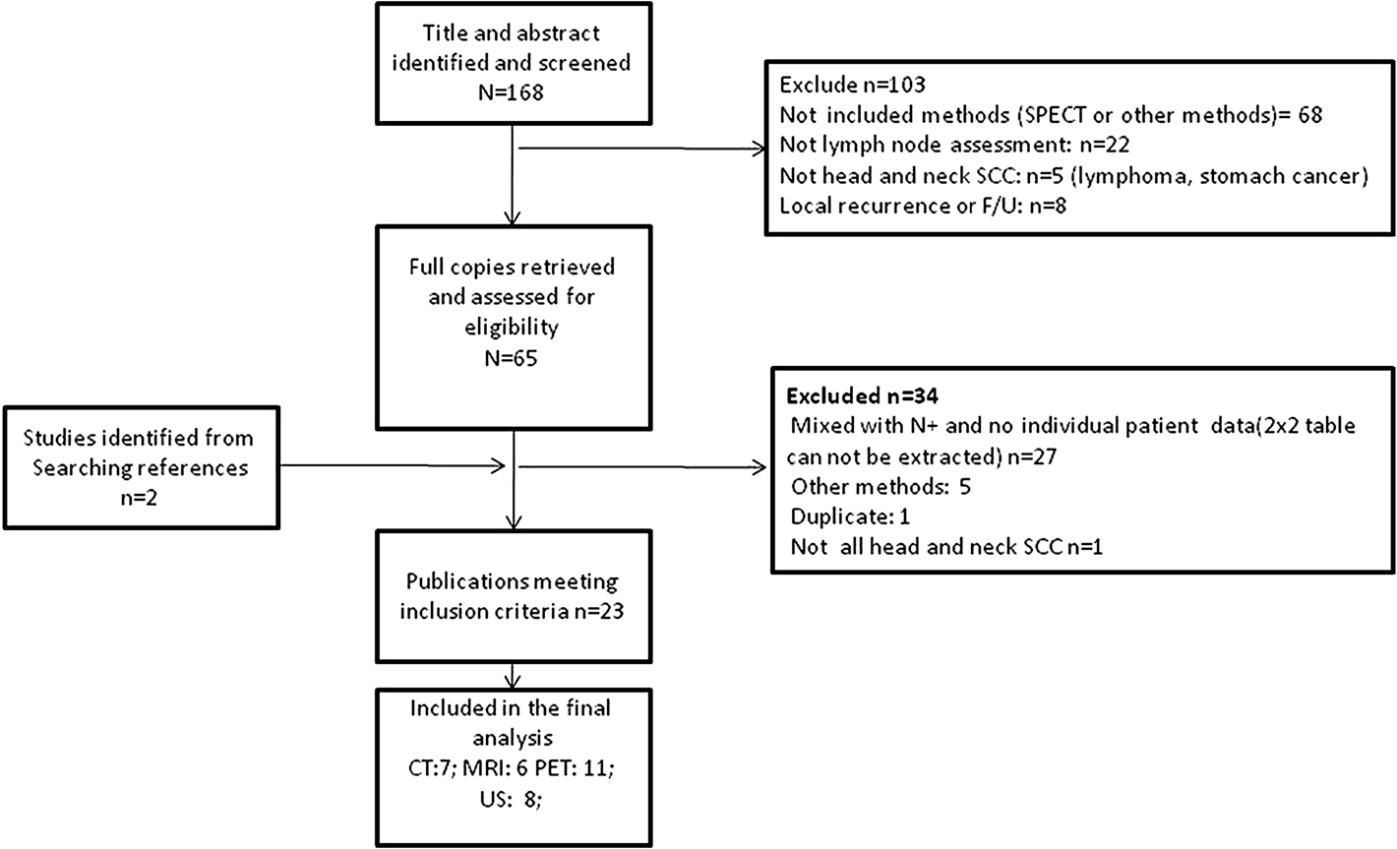
**Flow chart of the study selection process.** Of the 168 identified relevant articles, 7 studies fulfilled all inclusion criteria for CT, 6 studies for MRI, 11 studies for PET and 8 studies for US.

The abstracts and titles of 168 primary studies were identified for initial review based on the described search strategy. Full-text reviews were required for 65 publications to determine study eligibility. Subsequently, a total of 21 articles were selected based on agreement between the two reviewers. During the search process (Figure 1), we found that most studies were excluded due to a mix of cN0 and cN + patients without individual patient data for 2 × 2 tables. Seven studies fulfilled all inclusion criteria for CT, 6 studies for MRI, 11 studies for PET and 8 studies for US. The sensitivities and specificities with 95% confidence interval (CI) values and QUDAS scores for individual studies are summarized in Table 1.

**Table 1:** Summary of the included studies

**Author**	**year**	**Journal**	**Modality**	**TP**	**FP**	**FN**	**TN**	**Sensitivity (95%CI)**	**Specificity (95% CI)**	**QUADAS**
Stevens MH	1985	Arch Otolaryngol Head Neck Surg	CT	5	1	1	9	83%(54-100%)	90%(71-100%)	9
Maremonti P	1997	J Craniomaxillofac Surg	CT	6	0	6	12	50%(22-78%)	100%	7
Righi PD	1997	Head Neck	CT	6	0	4	23	60%(30-90%)	100%	7
Byers RM	1998	Head Neck	CT	2	2	9	12	18%(0-41%)	86%(67-100%)	5
Myers LL	1998	Laryngoscope	CT	4	1	3	9	57%(21-94%)	90%(71-100%)	10
Takes RP	1998	Int J Radiat Oncol Biol Phys	CT	14	3	12	35	54%(35-73%)	92%(84-100%)	11
Akoglu E	2005	J Otolaryngol	CT	2	1	1	10	67%(13-100%)	91%(74-100%)	10
Wilson GR	1994	Br J Plast Surg	MRI	11	14	0	13	100%	48%(29-67%)	11
Braams JW	1995	J Nucl Med	MRI	3	2	2	5	60%(17-100%)	71%(38-100%)	7
Maremonti P	1997	J Craniomaxillofac Surg	MRI	4	4	8	8	33%(7-60%)	67%(40-93%)	7
Yucel T	1997	Acta Radiol	MRI	4	1	1	12	80%(45%-100%)	92%(78-100%)	10
Akoglu E	2005	J Otolaryngol	MRI	2	1	1	10	67%(13-100%)	91%(74-100%)	10
Thomsen JB	2005	Acta Radiol	MRI	5	4	9	52	36%(11-61%)	93%(86-100%)	7
Braams JW	1995	J Nucl Med	PET	5	2	0	5	100%	71%(38-100%)	7
Myers LL	1998	J Otolaryngol	PET	7	0	0	4	100%	100%	10
Myers LL	1998	Laryngoscope	PET	7	0	2	15	78%(51-100%)	100%	10
Kau RJ	2002	Arch Otolaryngol Head Neck Surg	PET	1	1	4	6	20%(0-55%)	86%(60-100%)	13
Brouwer J	2004	Eur Arch Otorhinolaryngol	PET	2	1	1	11	67%(13-100%)	92%(76-100%)	9
Akoglu E	2005	J Otolaryngol	PET	2	1	1	10	67%(13-100%)	91%(74-100%)	10
Schoder H	2006	J Nucl Med	PET	6	4	3	23	67%(36-98%)	85%(72-99%)	10
Wensing BM	2006	Laryngoscope	PET	3	4	5	16	38%(4-71%)	80%(63-98%)	11
Iyer NG	2010	Head Neck	PET	15	4	14	113	52%(34-70%)	97%(93%-100%)	12
Liao CT	2010	J Nucl Med	PET	26	66	33	143	55%(31-57%)	68%(62-75%)	13
Richard C	2010	Arch Otolaryngol Head Neck Surg	PET	7	5	1	8	88%(65-100%)	62%(35-88%)	11
van den Brekel MW	1993	Eur Arch Otorhinolaryngol	US	21	13	15	39	58%(42-74%)	75%(63-87%)	9
Maremonti P	1997	J Craniomaxillofac Surg	US	8	1	4	11	67%(40-93%)	92%(76-100%)	7
Righi PD	1997	Head Neck	US	6	5	4	18	60%(30-90%)	78%(61-95%)	7
Byers RM	1998	Head Neck	US	3	4	8	10	27%(1-54%)	71%(48-95%)	5
Takes RP	1998	Int J Radiat Oncol Biol Phys	US	18	11	8	27	69%(52-87%)	71%(57-86%)	11
Hodder SC	2000	Br J Oral Maxillofac Surg	US	9	4	3	17	75%(51-100%)	81%(64-98%)	7
Akoglu E	2005	J Otolaryngol	US	3	4	0	7	100%	64%(35-92%)	10
Thomsen JB	2005	Acta Radiol	US	13	10	2	55	87%(70-100%)	85%(76-93%)	7

TP: true positive; FP: false positive; FN: false negative; FP: false positive.

QUADAS: Quality Assessment of Diagnostic Accuracy Studies.

Sensitivities and specificities with 95% confidence interval (CI) values were reported for individual studies.

**Figure 2:**
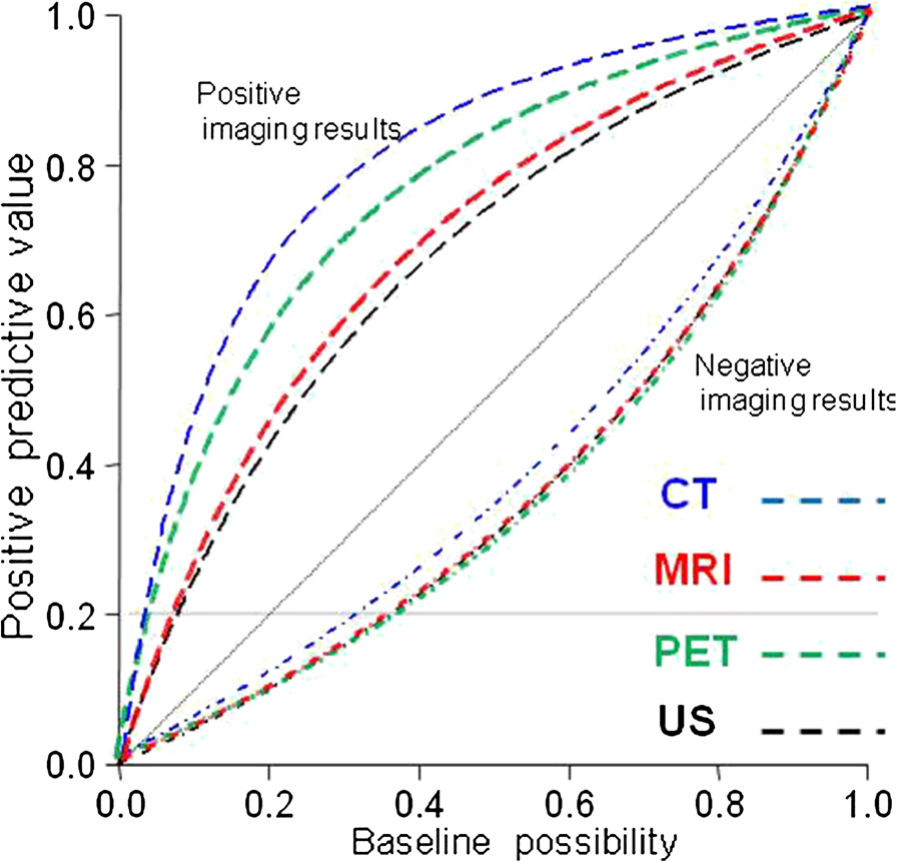
The positive predictive value generated based on Bayesian theory and the collected data.

There was no difference in the pooled sensitivity among these imaging modalities. The pooled estimates (Table 2 and Figure 2) for sensitivity were 52% (95% confidence interval [CI], 39% ~ 65%), 65% (34 ~ 87%), 66% (47 ~ 80%) and 66% (54 ~ 77%) on a per-neck basis for CT, MRI, PET and US, respectively. CT was superior to US in specificity, but there was no difference among CT, MRI and PET. The pooled estimates for specificity were 93% (87% ~ 97%), 81% (64 ~ 91%), 87% (77 ~ 93%) and 78% (71 ~ 83%) for CT, MRI, PET and US, respectively. There was no difference in QUADAS scores between the studied modalities (p > 0.05).

**Table 2:** The pooled estimates of different imaging modalities in cN0 neck evaluation

**Modalities**	**Sensitivity** **(95%CI)**	**Specificity (95%CI)**	**LR +** **(95%CI)**	**LR-** **(95%CI)**	QUADAS score* (95%CI)
**CT**	0.52 (0.39 ~ 0.65)	0.93 (0.87 ~ 0.97)	7.9 (3.6 ~ 17.4)	0.51 (0.38 ~ 0.68)	8.1 (3.8 ~ 12.4)
**MRI**	0.65 (0.34 ~ 0.87)	0.81 (0.64 ~ 0.91)	3.4 (1.8 ~ 6.2)	0.44 (0.21 ~ 0.93)	7.6 (4.1 ~ 11.1)
**PET**	0.66 (0.47 ~ 0.80)	0.87 (0.77 ~ 0.93)	5.2 (2.6 ~ 10.4)	0.39 (0.24 ~ 0.65)	10 (6.9 ~ 13.1)
**US**	0.66 (0.54 ~ 0.77)	0.78 (0.71 ~ 0.83)	3.0 (2.1 ~ 4.2)	0.44 (0.3 ~ 0.64)	7.5 (3.6 ~ 11.4)

*Yes: 1; No/unclear: 0.

LR+: likelihood ratio positive; LR-: likelihood ratio negative.

**Figure 3:**
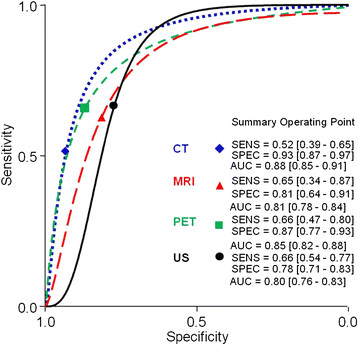
**The performance of different imaging modalities shown with summary receiver operating characteristic curves.** The pooled estimates for sensitivity were 52%, 65%, 66% and 66%, on a per-neck basis for CT, MRI, PET and US. The pooled estimates for specificity were 93%, 81%, 87%, and 78% for CT, MRI, PET and US, respectively.

Based on Bayesian theory and collected data with different pre-examination probabilities, the post examination positive and negative neck nodal probabilities were simulated (Table 3 and Figure 3). With pre-examination nodal metastasis set at 10%, 20% and 30%, the post-exam positive nodal metastasis probabilities were 47%, 66% and 77% for CT; 27%, 46% and 59% for MRI; 36%, 56% and 69% for PET; and 25%, 42% and 56% for US, respectively. The negative nodal metastasis probabilities were 95%, 89% and 82% for CT; 95%, 90% and 84% for MRI; 96%, 91% and 86% for PET; and 95%, 90% and 84% for US, respectively.

**Table 3:** The positive and negative predictive value of nodal metastasis following imaging exams among various baseline possibilities of neck nodal metastasis

**Imaging Modalities**	**Baseline possibility of neck nodal metastasis**	Positive predictive value*	**Negative predictive value**^&^
**CT**	10%	**47%**	95%
	20%	66%	89%
	30%	77%	**82%**
**MRI**	10%	**27%**	95%
	20%	46%	90%
	30%	59%	**84%**
**PET**	10%	**36%**	96%
	20%	56%	91%
	30%	69%	**86%**
**US**	10%	**25%**	95%
	20%	42%	90%
	30%	56%	**84%**

*: Possibility of neck nodal metastasis following a “positive” imaging result.

^&^: Possibility of “absent” neck nodal metastasis following a “negative” imaging result.

## Discussions

In the development of treatment paradigms for cN0 neck, it is important to be aware that most patients with a cN0 neck presented no cancer cells in the cervical lymph nodes and that over-treating the neck should be avoided.

Systematic review and meta-analysis of the accuracy of diagnostic tests can answer some clinically relevant questions, highlight important gaps in the evidence and aid in the design of further studies. Our results are the first to use meta-analytic data in neck metastasis rate estimation on different pre-test probabilities among different diagnostic imaging techniques. Because most individual studies have a limited number of cases, meta-analysis uses more data and provides more reliable results.

The optimal method for managing cN0 neck in SCC of the head and neck remains controversial. In 1994, Weiss et al. [3] recommended with decision analysis that when the probability of occult cervical metastases is more than 20% (with a positive predictive rate above 20%), the neck should be electively treated. Based on Bayesian theory, the predictive probability of neck nodal metastasis given that a test is negative or positive depends on the pre-test probability (baseline possibility), and the sensitivity and specificity of the test. According to our results, if the pretest prevalence (baseline possibility) of clinical occult neck metastases was set at 30%, the post-test negative predictive rate with negative CT, MRI, PET, and US results increased to 82%, 84%, 86% and 84%, respectively (with a positive neck lymph node metastasis rate below 20%), meaning a “watchful waiting policy” is possibly justified in these cases. In a recent report [8], the threshold was estimated even higher (44.4%) for oral tongue cancer. The occult cervical lymph node metastasis rate has been estimated at 25% ~ 35%, except for the glottic larynx, by palpation [9]. Therefore, a “watchful waiting policy” is feasible for some low pre-test occult metastases in cN0 neck patients, such as clinically T1 ~ 2N0M0 lip cancer patients. However, some clinically T4N0 tongue or tongue base cancers may have pre-test probability >60%. Even with a negative imaging result, the post-test probability is still approximately 20% (Figure 2), and elected neck dissection is still necessary for these patients. In patients with positive imaging results, even with a very low pretest possibility set at 10%, the positive nodal metastasis probabilities were all above 20% (47%, 27%, 36% and 25% for CT, MRI, PET and US, respectively). Elective neck dissection should be performed for all patients with positive pre-op diagnostic results.

According to our results, the pooled estimates for sensitivity were 52% (95% confidence interval [CI], 39% ~ 65%), 65% (34 ~ 87%), 66% (47 ~ 80%) and 66% (54 ~ 77%) on a per-neck basis for CT, MRI, PET and US, respectively. The pooled estimates for specificity were 93% (87% ~ 97%), 81% (64 ~ 91%), 87% (77 ~ 93%) and 78% (71 ~ 83%) for CT, MRI, PET and US, respectively. Our results are similar to a previous meta-analysis [5], which compared PET to other traditional imaging modalities (including CT, MRI and US-guided fine-needle aspiration). This previous study concluded that PET was not superior to other imaging modalities in a cN0 neck work-up. However, the study was focused on all nodal statuses and subgroup analysis for N0 patients combined CT, MRI and US-guided fine-needle aspiration (US-FNA) in the same group.

PET exam is the more expensive imaging option for nodal surveillance; however, it did not provide better sensitivity and specificity. Therefore, it should not be routinely used in neck nodal status work-ups. In our opinion, CT or MRI is preferred for cN0 neck pre-op evaluation because CT and MRI had similar diagnostic sensitivities to PET and US. Furthermore, CT and MRI can evaluate the status of primary tumor at the same time. The US is an inexpensive and convenient tool to monitor nodal status and can be used with real-time guided fine-needle aspiration. However, the primary tumor lesion and some deep-seated lymph nodes, such as retropharyngeal nodes, cannot be assessed [10].

The alignment of the results between preoperative imaging and histologic specimen after neck dissection should be taken into consideration. According to previous reported literatures [11,12], the rate of regional recurrence in pN0 patients varying from 3% to 10%. Applying only the histopathological results as reference standard, one could underestimate the real occult metastasis rate. Therefore, we included studies using either pathological examinations, or clinical follow-up results, or both as references. Besides, the criteria for positive results in pre-operative diagnostic imaging were not uniform in different institutions, and may be operator-dependent. These variations all leaded to heterogeneity in this meta-analysis, and this was the reason to adapt a random effect model for data pooling.

During this review, we found that US-FNA had a very high specificity; if the US-FNA cytology had a positive result, almost all of the histology specimen results also proved positive [1,13-15]. Therefore, in our opinion, US is preferred for neck status follow-up in “watchful waiting” patients, and US-FNA can be performed if nodal metastasis is suspected.

In our review, we did not include US-FNA because US-FNA cytology examination required a cytologist’s assistance and was used after US examination. However, it must be noted that US-FNA had 100% specificity because there were no false-positive cases. Therefore, we did not believe that the comparison of sensitivity, specificity and summary ROC curves were justified between US-FNA and other imaging modalities because the lack of false-positive cases spuriously inflates the value of the area under the ROC curve.

In our review and subsequent meta-analysis, we found modern imaging modalities had fair diagnostic performance in cN0 neck patients. For positive imaging results, elective neck dissection is indicated; for some select low-risk patients with pre-test probability below 30% of nodal metastasis, a “watchful waiting policy” may be an acceptable alternative to neck dissection if strict adherence to a cancer surveillance protocol is followed.

## Conclusions

Modern imaging modalities offer similar diagnostic accuracy to define and diagnose cN0 neck. Minimizing morbidity and avoiding elective neck dissection is acceptable in some select cases.

## Competing interests

We declare no conflicts of interest.

## Authors' contributions

LJL: conception and design, WCL: acquisition of data (reviewer 1), WLH: statistical analysis, CTW: acquisition of data (reviewer 2), MSL: Instructor (revising). All authors read and approved the final manuscript.

## Pre-publication history

The pre-publication history for this paper can be accessed here:

http://www.biomedcentral.com/1471-2407/12/236/prepub
